# Unmasking lymphoma immune reconstitution inflammatory syndrome in a patient with pyrexia of unknown origin: a case report

**DOI:** 10.1186/s43046-020-0019-7

**Published:** 2020-01-30

**Authors:** Mansi Mahajan, Bhanu Prasad Venkatesulu, Omar Sallam, Kanika Taneja, Megan Scott, Indira Brar

**Affiliations:** 1 School of Medicine, Wayne State University, Detroit, MI USA; 2 Department of Internal Medicine, Henry Ford Hospital, Detroit, MI USA; 3 Department of Pathology, Henry Ford Hospital, Detroit, MI USA

**Keywords:** HIV, IRIS, PUO, Lymphoma

## Abstract

**Background:**

Immune reconstitution inflammatory syndrome (IRIS) is a constellation of inflammatory disorders that are unmasked after the initiation of anti-retroviral therapy (ART) in Human immunodeficiency virus (HIV) infected patients. Unmasking lymphoma IRIS is a relatively rare manifestation after initiation of anti-retroviral therapy.

**Case presentation:**

We report a 44-year-old male with HIV on 4 months of ART presenting with pyrexia of unknown origin with a diagnosis of unmasking Hodgkin’s lymphoma IRIS stage IV with B symptoms. This case portrays the importance of recognizing the possibility of Hodgkin’s lymphoma as a possible manifestation of IRIS within the first 6 months of initiation of ART.

**Conclusion:**

Patients presenting with pyrexia of unknown origin and lymphadenopathy within the first 6 months of initiation of ART, lymphoma diagnosis should be on the high threshold of suspicion as portrayed by our case.

## Background

Immune reconstitution inflammatory syndrome (IRIS) is a constellation of inflammatory disorders that are unmasked after the initiation of anti-retroviral therapy (ART) in Human immunodeficiency virus (HIV) infected patients. Only 10–15% of patients develop IRIS after initiation of ART and the risk factors are low CD4 count, high HIV RNA viral load at baseline with uptrend of CD4 count and downtrend of HIV viremia after starting ART [1]. The typical manifestation are of infectious etiology like *Mycobacterium tuberculosis*, *Mycobacterium avium* complex, cytomegalovirus, *Cryptococcus neoformans*, Pneumocystis jiroveci, Herpes simplex virus, and hepatitis B virus [2, 3]. The Swiss cohort study had reported that the Kaposi sarcoma tends to manifest the highest during the first 6 months of starting ART therapy. This led to the possible hypothesis that malignancy may also be an unmasking manifestation of IRIS syndrome [4]. The Center for AIDS Research (CFAR) Network of Integrated Clinical Systems (CNICS) defined unmasking lymphoma IRIS as a manifestation of Hodgkin’s lymphoma or non-Hodgkin’s lymphoma within the first 6 months of initiation with HIV viremia suppression more than ≥ 0.5 log reduction in HIV RNA log10 copies/mL. In comprehensive analysis of 27,000 patients over a span of 15 years the CNICS group identified 482 patients with a diagnosis of lymphoma of whom 56 (12%) fulfilled the criteria of unmasking lymphoma IRIS. Among them, the most common diagnosis were diffuse large B cell lymphoma (DLBCL) 22 (39%), Hodgkin lymphoma (HL) 12 (21%), 10 (18%) primary central nervous system lymphoma, Burkitt lymphoma 5 (9%), and other non-Hodgkin lymphoma 7 (13%) [5]. Herein, we report a HIV positive patient who presented with pyrexia of unknown origin and was eventually diagnosed with an unmasking Hodgkin’s lymphoma IRIS after 4 months of initiation of ART.

## Case presentation

**Fig. 1:**
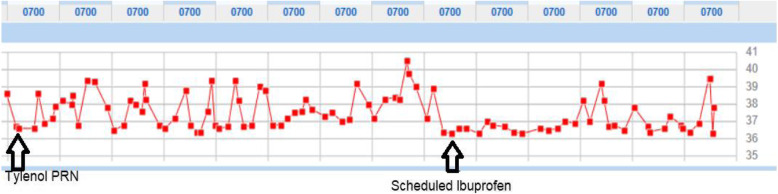
The pattern of fever patient exhibited when he was admitted to the hospital as inpatient

**Fig. 2:**
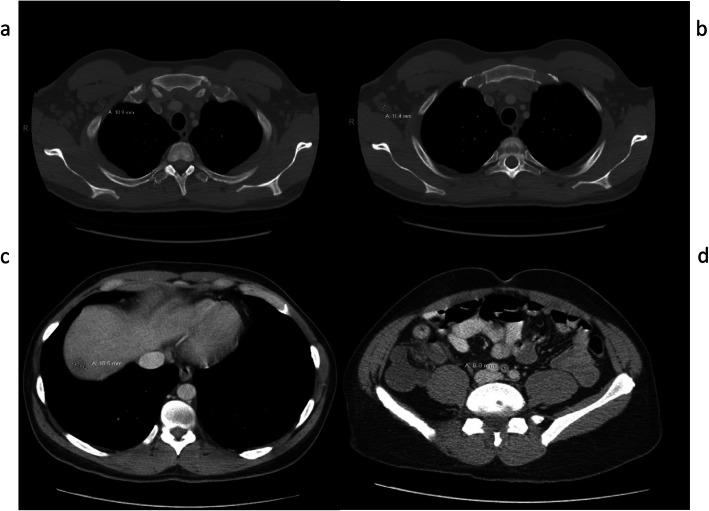
Computed tomography of chest (**a**, **b**), abdomen (**c**), and pelvis (**d**) showing mild right axillary, with moderate right pelvic lymphadenopathy with largest lymph node measuring up to 1.8 cm short axis adjacent to the distal right external iliac vasculature. There is diffuse heterogeneous enhancement of the hepatic parenchyma with scattered nodular regions of hypoattenuation measuring up to 1.9 cm in size

A 44-year-old male presented to our infectious diseases department with complaints of nausea and fever for 4 days. He also endorsed night sweats without loss of weight. The patient’s past medical history was significant for HIV diagnosed 4 months prior to the current clinical presentation (Fig. 1). His current medications include darunavir-Cobicistat, emtricitabine, and tenofovir for HIV and sulfamethoxazole with trimethoprim for pneumocystis carni pneumonia prophylaxis. General clinical examination showed a general condition of Eastern Co-operative Oncology Group Performance status 1. Examination of his neck showed enlarged, rubbery, matted bilateral level 1b and level 2 neck lymph nodes, and enlarged, rubbery, matted right axillary lymph nodes. His baseline CD4 count was 163 and his current CD4 count after ART for 4 months was 149. His baseline HIV RNA was 18,355 and his current HIV RNA after ART for 4 months was less than 20 copies. He had comprehensive pyrexia of unknown origin workup which was negative. Computed tomography of neck, abdomen, and pelvis showed enlarged bilateral neck nodes in level 1b, level II, mild right axillary, mediastinal and hilar lymphadenopathy, moderate right pelvic lymphadenopathy, mild periportal lymphadenopathy is present with diffuse heterogeneous enhancement of the hepatic parenchyma. His serum LDH was 209. Since his extensive workup was negative for infectious etiology, we did an excisional biopsy of his axillary lymph node. The histopathology revealed classic Hodgkin lymphoma nodular sclerosis type positive for CD45 and CD5. He underwent a bone marrow biopsy which was positive for bone marrow involvement and negative for FLT3 mutation analysis assessed by internal tandem duplication. The patient was diagnosed with an unmasking Hodgkin’s lymphoma IRIS stage IV with B symptoms and planned for chemotherapy with ABVD regimen. In view of possible interaction of his ART with chemotherapy, his HIV medications were changed to bictegravir, emtricitabine, and tenofovir alafenamide (Fig. 2).

## Discussion

**Fig. 3:**
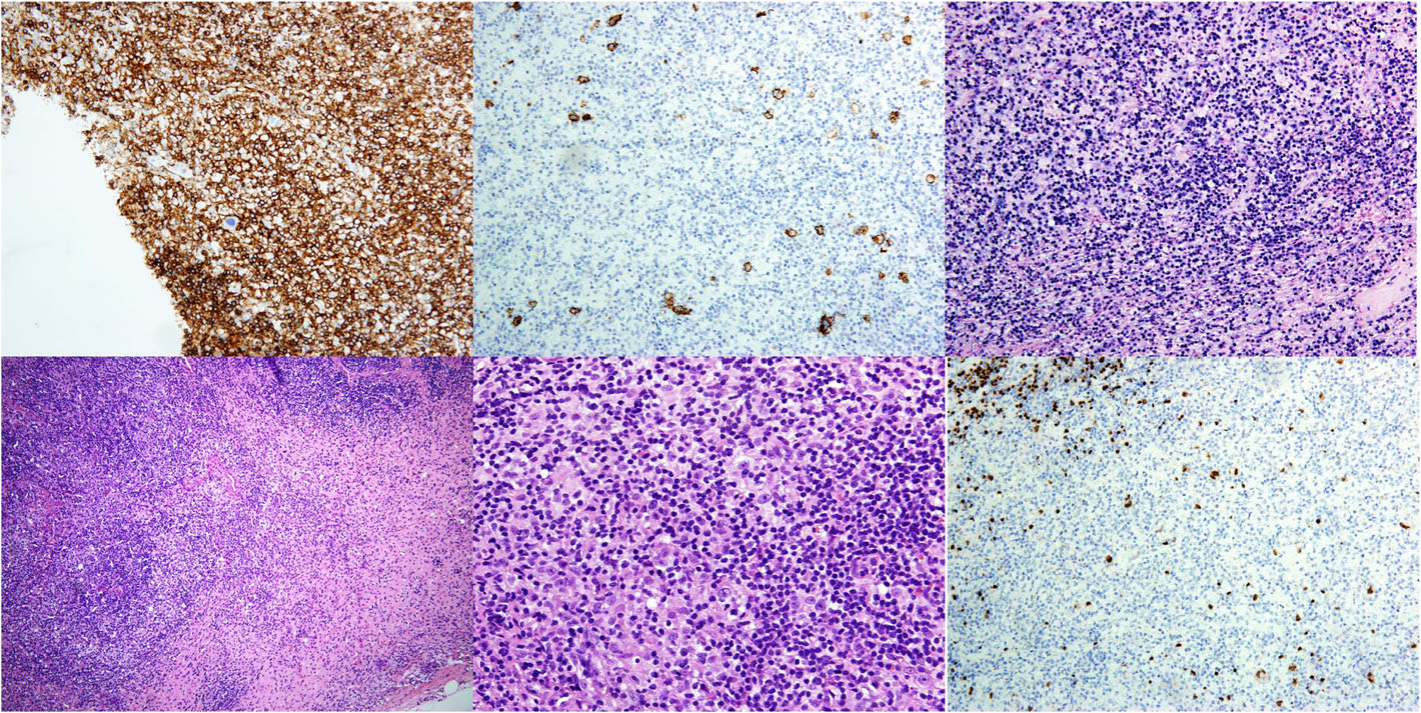
Sections demonstrate atypical nodular lymphoid infiltrate separated by ill-defined fibrotic bands. The nodular lymphoid infiltrate is mainly composed of large atypical cells morphologically resembling Reed-Sternberg cells, in a mixed inflammatory background consisting of small lymphocytes, histiocytes, plasma cells, and occasional eosinophils. The large atypical cells are positive for CD30, PAX5 (dim) and negative for CD20, CD15, CD45, and CD3. CD3 stains numerous T cells in the background. EBER-ish is positive in the Hodgkin cells based on EBV stain performed on with appropriate controls

HIV predisposes to a spectrum of malignant disorders in view of suppression of the immune system. The AIDS cancer match study group reported that in people with AIDS, Kaposi’s sarcoma (KS) was the most commonly diagnosed malignancy followed by non-Hodgkin lymphoma (NHL). HIV increased incidence of KS by 310-fold, NHL by 113-fold and Hodgkin’s disease by 7·6-fold [6]. Yanik et al. reported that the timing of cancer incidence after ART was distinct among various cancer types with KS having the highest incidence in the first 6 months after ART initiation (IR, 1342 cases per 100,000 person-years [95% CI, 1071–1683 cases per 100,000 person-years]), followed by lymphomas (both Hodgkin and non-Hodgkin) (IR, 660 per 100,000 person-years [95% CI, 479–912 cases per 100,000 person-years]) with the possibility that the immune changes that occur with ART initiation might be responsible for unmasking of malignancies as a IRIS manifestation [7]. Lanoy et al. reported that in a cohort of 64,368 HIV patients 187 patients were diagnosed with HL. They found that risk of HL was related to the timing of ART initiation with the highest risk in the first 3 months (RR 2.95, CI 1.64–5.31) with a slightly lowered risk between 4 and 6 months (RR 1.63) and normal risk (RR 1.00) after 6 months. CD4 count of 50–99 CD4 cells/mm^3^ had the highest risk of HL. Our patient was in the group of 4–6 months with a relatively higher CD4 count of 149 [8]. Kowalkowski et al. reported the Veterans Affairs HIV Clinical Case Registry incidence rate of 6.8/10,000 person-years for HL with HIV. They also reported that the rate of rise of CD4 count rise was worst among HL patients compared to other HIV patients with cancer. The HL risk was also lowest if the patients had > 80% time undetectable HIV RNA. Our patient had atypical presentation in this aspect since he had less than 20 copies of HIV RNA detected and had relatively stable CD4 count compared to the baseline CD4 count [9]. Gotti et al. reported similar trend in that the incidence rate was 9.9 (95% CI, 6.7–14.1) per 10,000 person-years of follow-up. They also reported that the risk of HL is highest with low CD4 count at diagnosis and within first 6 months of initiation of ART therapy [10]. The HL survival outcomes are not different in patients with IRIS vs non-IRIS presentation. The treatment goals are similar to other HL patients without HIV (Fig. 3).

## Conclusion

Patients presenting with pyrexia of unknown origin and lymphadenopathy within the first 6 months of initiation of ART, lymphoma diagnosis should be on the high threshold of suspicion as portrayed by our case.

## Data Availability

Not applicable.

## Abbreviations

ART: Anti-retroviral therapy
CFAR: Center for AIDS Research
CNICS: Network of Integrated Clinical Systems
DLBCL: Large B cell lymphoma
HIV: Human immunodeficiency virus
HL: Hodgkin lymphoma
IRIS: Immune reconstitution inflammatory syndrome
